# The “Texas Longhorn” Syndrome: A Case of Posterior Interosseous Nerve Palsy With Noteworthy Observations

**DOI:** 10.7759/cureus.12760

**Published:** 2021-01-18

**Authors:** Hassan Kesserwani

**Affiliations:** 1 Neurology, Flowers Medical Group, Dothan, USA

**Keywords:** peripheral nerve disorders, radial nerve injury

## Abstract

The posterior interosseous nerve (PIN) is quite a fascinating nerve. It wraps around the radial neck akin to the fibular nerve wrapping around the fibular neck. It can be compressed by the arcade of Frohse in a fashion similar to median nerve entrapment by the carpal tunnel. Furthermore, it divides into a number of branches, a simulacrum of the cauda equina, as it emerges from the supinator muscle. It is also associated with space-occupying pathologies; lipomas, and ganglion cysts being among the most common. It also has other interesting anatomical properties, such as fascicular compartmentalization, explaining PIN paralysis with radial nerve injuries. Finally, PIN lesions can lead to partial paralysis of finger extensors leading to the "Texas longhorn" hand gesture. We seize upon a case of PIN entrapment by fibrous adhesions and the arcade of Frohse, and explore the functional anatomy of the PIN.

## Introduction

At the radiocapitellar joint of the elbow, the radial nerve bifurcates into the posterior interosseous nerve (PIN) and the superficial radial nerve (SRN). The PIN passes between both heads of the supinator muscle and wraps around the radial neck, in a manner reminiscent of the fibular nerve wrapping around the fibular neck. In 25% of cases, the PIN contacts the neck of the radius directly over the aptly named "bare area" of the radial neck. Here the PIN supplies the supinator muscle and the extensor carpi radialis brevis (ECRB). As it emerges from the supinator, the PIN divides into multiple divisions resembling a mini-cauda equina. These branches supply the extensor group of muscles of the forearm; extensor digitorum communis (EDC), extensor digiti quinti (EDQ), extensor carpi ulnaris (ECU), abductor pollicis longus (APL), extensor pollicis longus (EPL), extensor pollicis brevis (EPB), and extensor indicis proprius (EIP) [1]. These muscles extend the digits at the metacarpophalangeal joints, ulnar-extend the wrist and extend and abduct the thumb in the plane of the palm, as the names imply. In summary, the PIN supplies all the extensor muscles of the forearm except the extensor carpi radialis longus (ECRL), the radial extensor deviator of the wrist, and the Brachioradialis (BR), the elbow flexor with the forearm in 90-degree pronation. 

It immediately follows that the PIN does not supply the BR, ECRL, and of course, the triceps, all of which are supplied by the radial nerve proper. Furthermore, the PIN is a purely motor nerve. Therefore, with PIN injury, radial deviation of the wrist and sensation over the SRN distribution are preserved, using useful clinical pearls.

In 30% of cases, the PIN pierces the superficial head of the supinator and enters an inverted fibrous arch, shaped like a parabola. This is the arcade of Frohse which attaches like a sling from the tip to the medial edge of the lateral epicondyle. The thickness and size of the opening of this arcade are highly variable. The arcade of Frohse is membranous in 68% and tendinous in 32% of cases [2]. This arch may press on the PIN in a manner akin to the compression of the median nerve at the carpal tunnel. These three aforementioned anatomic parallels are a useful aid in reminding the clinician of the neuroanatomy of the PIN. 

In their 20-year retrospective series of 18 cases, Quignon et al., stress the pattern of finger extension paresis (finger drop) plus radial deviation of wrist extension (due to sparing of the ECRL). Most of their patients were male manual workers. Most cases had a tumor as an etiology, lipoma being the commonest. This was followed by trauma, iatrogenic or idiopathic with equal frequency. Interestingly, in seven cases, entrapment was caused by a sharp tendon of the ECRB. Treatment was frequently done by neurolysis and the prognosis was generally good. Better outcomes were associated with a shorter duration of paresis [3]. The PIN syndrome is also referred to as the supinator syndrome or the radial tunnel syndrome, with subtle distinctions, which we will outline in the Discussion section.

## Case presentation

We present the case of a 62-year-old right-handed man who presents with vague aching pain of the right upper forearm. The weakness of grip progressed over the course of two weeks and the decline halted. In particular, he noted difficulty elevating the ring and middle fingers of the right hand. He denied any radicular symptoms, but noted visible atrophy of the extensor group of muscles of the right forearm. Numbness and tingling were denied. There was no reported trauma.

His past medical history was unremarkable, with no prescribed medications.

**Figure 1:**
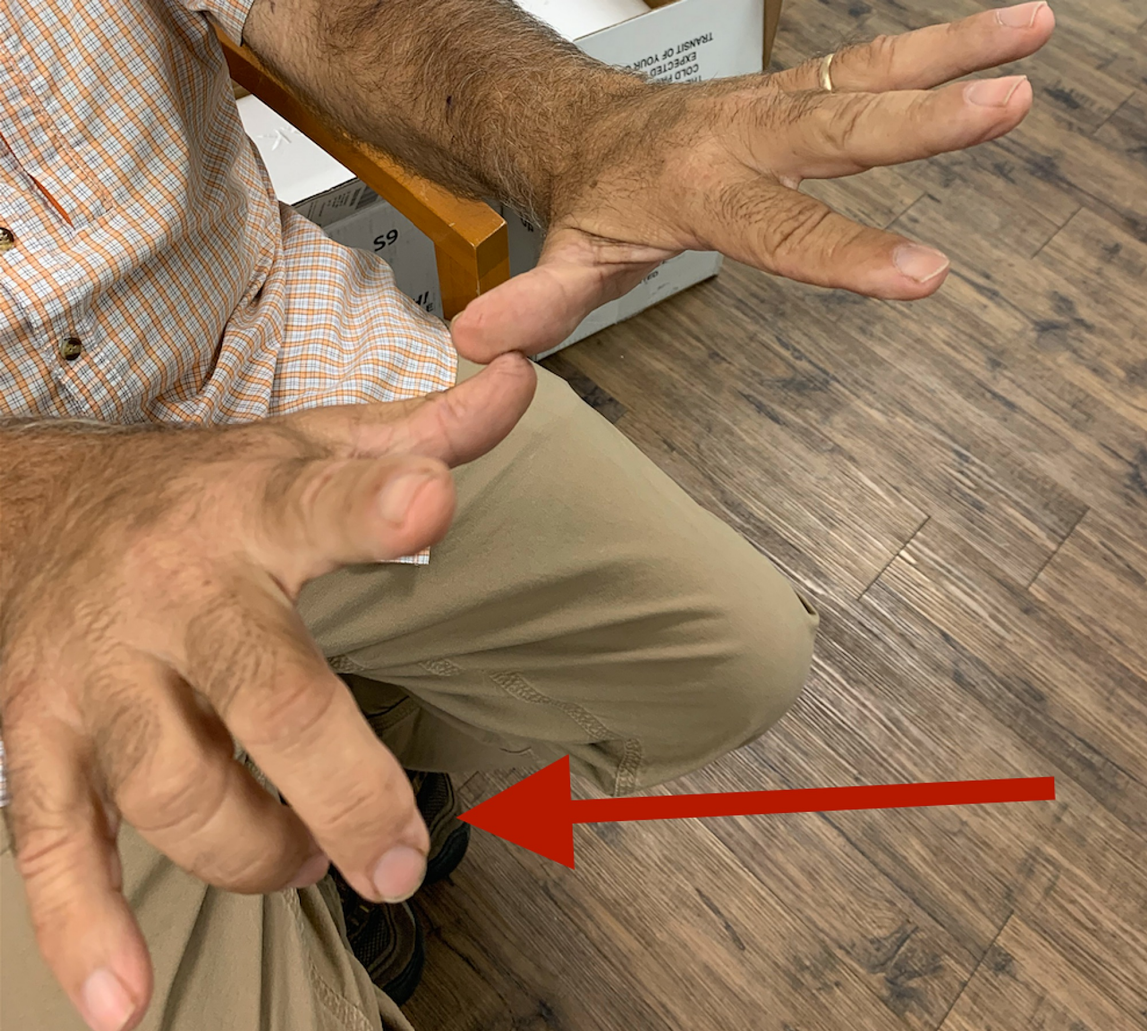
Texas longhorn: finger drop of right middle and ring fingers (red arrow), the classic manifestation of a posterior interosseous nerve palsy

The right arm neurologic examination was significant for atrophy of the extensor group of muscles of the right forearm and finger drop of the right middle and ring fingers, with mild weakness of index finger extension, the so-called "Texas longhorn" gesture (Figure 1).

There was also weakness of right thumb extension in the plane of the palm. Right wrist extension and distal interphalangeal extension of all the digits were normal. Deep tendon reflexes including the triceps, BR, and biceps were symmetric in both arms. Sensation over the right superficial radial sensory nerve was preserved to touch and pinprick. Tenderness was elicited by deep compression of the radial (supinator) tunnel over the right supinator muscle. This constellation of findings is highly typical of a right interosseous nerve lesion at the radial tunnel. Particular emphasis should be placed on the preservation of the right ECRL muscle (radially deviated wrist extension), triceps, and BR, all being radial nerve-innervated muscles.

**Figure 2:**
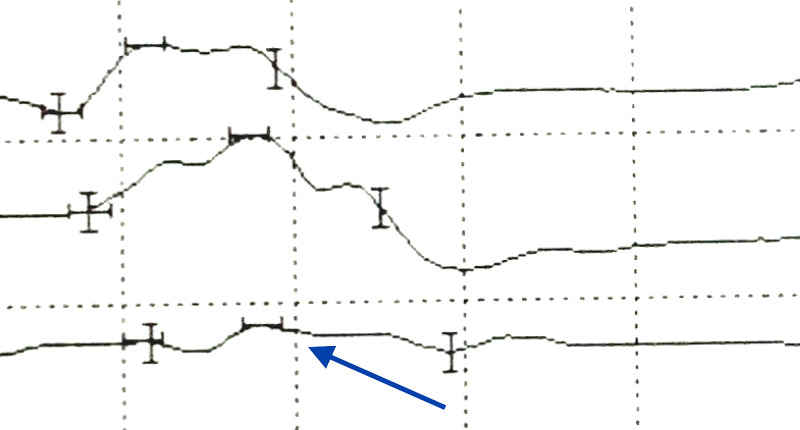
Nerve conduction study of posterior interosseous nerve (radial nerve) recording from the extensor indicis proprius; note conduction block with recording above the radial head (blue arrow).

A nerve conduction study (NCS) of the right radial motor nerve, recording from the EIP, revealed a conduction block above the right radial tunnel (Figure 2). 

**Figure 3:**
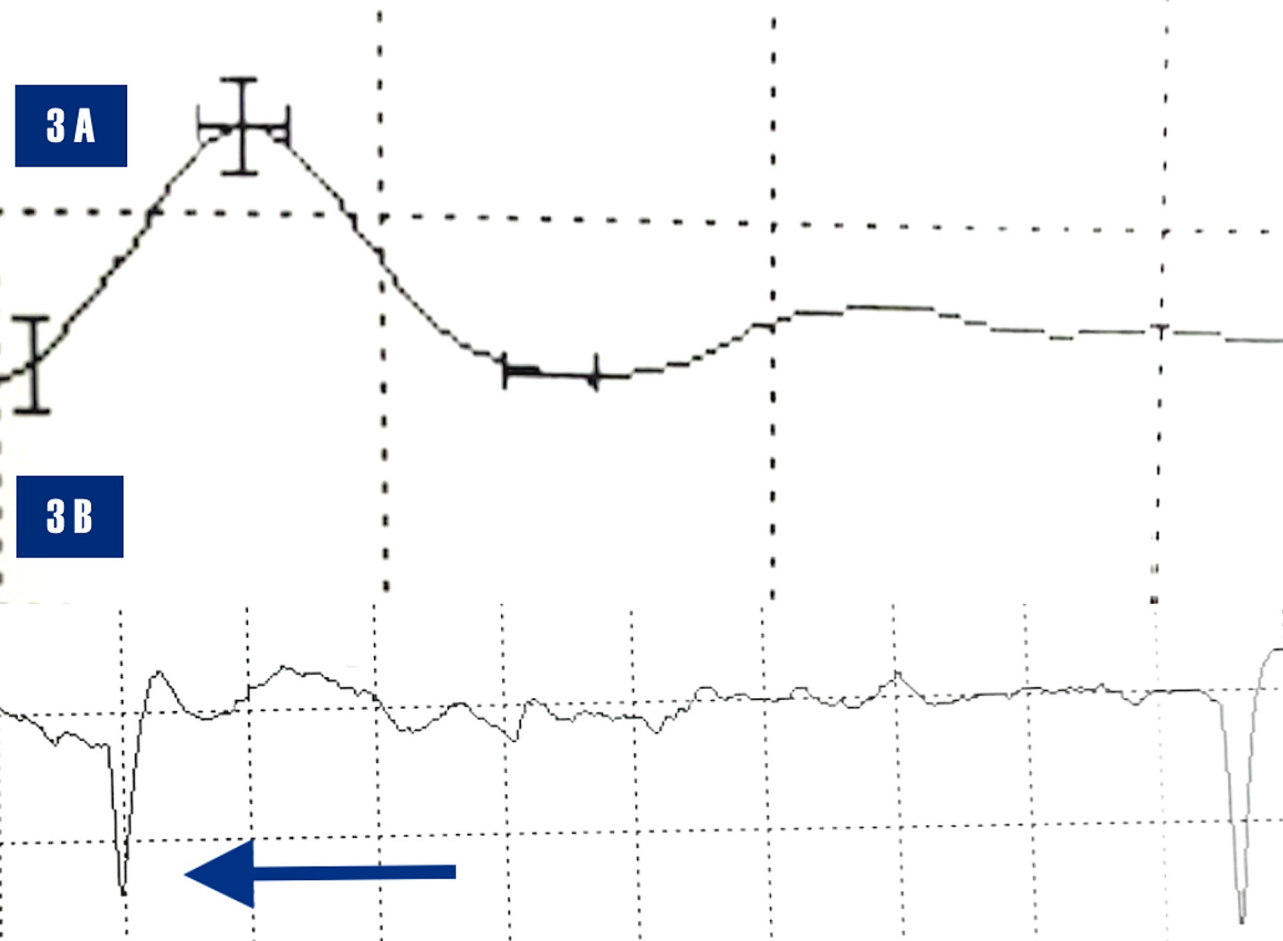
3A. The nerve conduction study demonstrates a normal right radial sensory amplitude of 12 microvolts (normal > 10 microvolts). 3B. Electromyogram of right extensor digitorum communis demonstrates acute denervation, positive waves (blue arrow)

The NCS of the right superficial radial sensory amplitude revealed a normal sensory nerve action potential (SNAP) amplitude, 12 microvolts (normal: greater than or equal to 10 microvolts), with acute denervation of the right EDC on electromyography (EMG) (Figure 3). 

**Figure 4:**
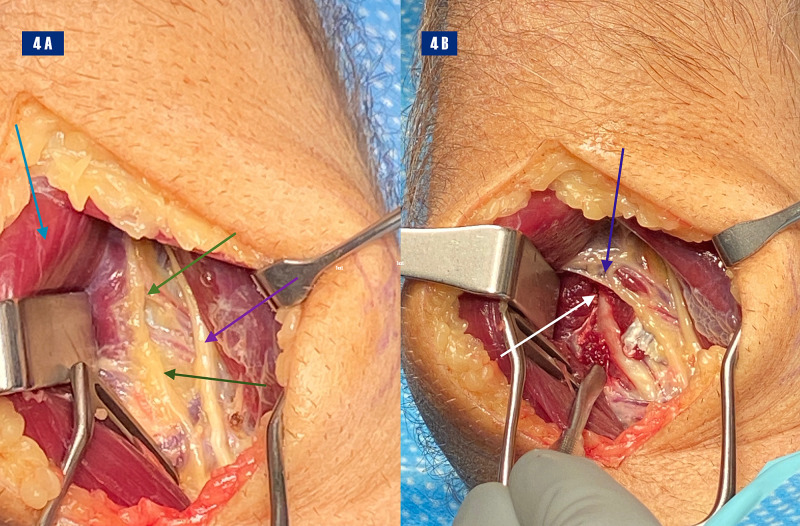
Right forearm incision: 4A: Deflection of right brachioradialis (light blue arrow), posterior interosseous nerve restrained by fibrous adhesions (green arrows), tendon of extensor carpi radialis brevis (purple arrow). 4B: Dissected arcade (blue arrow), posterior interosseous nerve (white arrow)

Surgical exploration of the right radial tunnel revealed a right PIN that was pinned down by the fascia and arcade of Frohse of the right supinator muscle. An incision was made along the dorsal border of the BR starting at the elbow crease and extending distally about 10 centimeters (cm). The lateral cutaneous nerve of the forearm and the fascial plane between the BR and the ECRL was identified. This was opened and the dissection was carried out deeply. The radial nerve was identified proximally and no adhesions noted, even proximal to the elbow. Distally, the SRN and PIN were identified. There was significant compression of the PIN by the arcade of Frohse at the proximal edge of the supinator. The compression was relieved by dissection and the PIN was tracked distally along the supinator to ensure no further bands of compression distal to the arcade (Figure 4).

One month after surgery, the patient noted a definite but small improvement in finger extension. He was advised that recovery is a slow process and may take up to 12 months. 

## Discussion

A fascinating retrospective review of 15 patients by Maldonado et al., recommended exercising caution and a careful reanalysis of PIN syndrome in nontraumatic cases. Four patients had a well-defined mass compressing the PIN. The rest of the patients had MRI and clinical findings, such as atrophy and weakness, NCS/EMG findings, and nerve hypertrophy beyond the territory of the PIN. These findings reappraised the diagnosis to a more diffuse process such as an inflammatory neuropathy, as seen with a mononeuritis multiplex or a posterior cord of brachial plexus. They highlighted the manifold pathophysiologic classification of PIN lesions to compression, inflammation, and fascicular constriction [4].

Vrieling et al., in a study of 14 patients, distinguished between PIN paralysis and a radial tunnel syndrome, the latter presenting with the typical pattern of weakness plus forearm pain. They recommended an observation period of six to eight months in those patients with PIN paresis, prior to exploration [5].

Anania et al., in a review of seven cases, emphasized that entrapment distal to the arcade of Frohse is rare, reported in only one out of their seven cases [6]. Berton et al. studied cadavers and looked at the pennation pattern of the superficial and deep heads of the supinator. The proximal arcade was tendinous in almost two-thirds of cadavers and the distal arcade was tendinous in about three-quarters of cadavers. In between, there were no adhesions or tendinous lesions [7].

In a retrospective review by Cravens and Kline of over 15 years, PIN disorders represented 32 out of 170 radial nerve disorders. The table below classifies the lesions (Table 1) [8].

**Table 1:** Table classifying PIN lesions in one of the largest retrospective studies. PIN: Posterior interosseous nerve

Lesion	Occurrence Number
Entrapment at the arcade of Frohse	14
Laceration	6
Fracture	6
Compression or contusion	3
Tumor	3

Twenty-six patients underwent surgery including neurolysis of adhesions, decompression, excision of the local tumor, and in four cases of laceration; end-to-end suture repair, and rarely grafts. The overall prognosis was very good, with near recovery in most cases.

There are several case reports of proximal radio-ulnar ganglion lesions involving the PIN with excellent recovery following excision [9], of intraneural PIN lipomas [10], isolated case reports of PIN schwannomas [11], neuralgic amyotrophy presenting as an isolated PIN palsy [12], and rheumatoid arthritis with synovial invasion [13].

An interesting and not unusual differential diagnosis of finger drop is due to a cervical C8 nerve root lesion. The rationale being that the EDC has nerve supply from the cervical C6, C7, and C8 nerve roots. In a report of 17 cases, Koda et al. note that recovery was usually delayed by at least nine months after surgery, and surgical decompression was favored over conservative management [14].

It is conceivable that the PIN and radial nerve proper (supplying BR, ECRL, and triceps) are compartmentalized. A high-resolution ultrasound study of the radial nerve at the spiral groove revealed a figure of eight fascicular pairs that split distally in the upper arm [15]. Interestingly, a prior study of 26 patients with radial nerve, PIN and posterior cord brachial plexus injuries due to trauma and tumor, had identified dual pathology in the radial nerve proper and PIN, mostly focal nerve hypertrophy [16]. 

Furthermore, the profile of radial nerve injuries is different in the pediatric population. In a retrospective study of 19 children with traumatic nerve injuries of the radial nerve, the lesion was localizable to the PIN in 37% of cases, an unusually high frequency [17].

Bäumer et al. point out that PIN syndrome can be due to trauma, tumor, compression, or inflammation. They also report that partial lesions of the proximal radial nerve may mimic a PIN syndrome. They deployed magnetic resonance neurography to study 19 patients with PIN syndrome and observed T2-weighted hyperintense signal abnormalities of the proximal radial nerve in 84.2% of patients, with the larger proportion of radial nerves revealing a partial fascicular lesion. These lesions occurred in the deeper branch of the radial nerve that continued into the PIN. Some of these lesions also extended proximally into the posterior cord of the brachial plexus, and this may explain how an immune-mediated brachial plexopathy can cause a PIN syndrome. They also posit that a PIN syndrome can occur secondary to a more proximally placed lesion due to a selective fascicular lesion, with the PIN occupying a dorsomedial location along the radial nerve. They also speculate on a potential explanation for the intriguing phenotype of a "Texas longhorn" hand gesture, with selective middle and ring finger drop due to a length-dependent polyneuropathy, noting that the middle and ring fingers are the longest [18]. 

## Conclusions

In summary, lesions of the PIN are quite variable and include tumors, trauma, inflammation, and compressive lesions. One can often obtain a "spot diagnosis" due to its unique clinical profile. Fascinating mimics include a C8 nerve root lesion or a high radial nerve lesion due to fascicular compartmentalization of the PIN and radial nerve proper. Its journey in the forearm is highlighted by useful landmarks including the radial neck, the proximal and distal arcades of the supinator muscle, where potential pathology may occur, as noted above.
